# Intimate Partner Violence: The Relationship Between the Stages of Change, Maintenance Factors, and the Decision to Keep or Leave the Violent Partner

**DOI:** 10.3390/jcm14020517

**Published:** 2025-01-15

**Authors:** Marisalva Fávero, Rita Oliveira, Amaia Del Campo, Amadeu Fernandes, Diana Moreira, Maria Dolores Lanzarote-Fernández, Valéria Sousa-Gomes

**Affiliations:** 1 Social and Behavioral Sciences Department, University of Maia, 4475-690 Maia, Portugal; ritaffoliveira@hotmail.com (R.O.); afernandes@umaia.pt (A.F.); vgomes@umaia.pt (V.S.-G.); 2 Center for Psychology, University of Porto-CPUP, 4200-135 Porto, Portugal; dimoreira@ucp.pt; 3 Faculty of Education, University of Salamanca, 37008 Salamanca, Spain; acampo@usal.es; 4 Faculty of Philosophy and Social Sciences, Centre for Philosophical and Humanistic Studies, Universidade Católica Portuguesa, 4710-362 Braga, Portugal; 5 Institute of Psychology and Neuropsychology of Porto–IPNP Health, 4000-055 Porto, Portugal; 6 Centro de Solidariedade de Braga/Projecto Homem, 4700-024 Braga, Portugal; 7 Personality, Psychological Evaluation and Treatment, University of Sevilla, 41004 Seville, Spain; lanzarote@us.es

**Keywords:** intimate partner violence, victims, maintenance factors, transtheoretical model of change, DVSA

## Abstract

**Objectives:** Violence in intimate relationships (IPV) is understood as one of the most common forms of violence, being maintained by cultural habits and customs, and legitimized from generation to generation. This study aims at a comprehensive review of the literature on IPV, the relationship between the stages of change, maintenance factors, and the decision to keep or leave the violent partner. **Methods:** A comprehensive literature search was conducted to identify journal articles focused on IPV, following online databases as well as a manual review from relevant peer-reviewed journals. **Results:** Seeking help is one of the main steps in the process of change, and the stages of change are directly related to the success of interventions, so identifying stages early provides a more appropriate and effective choice of intervention. **Conclusions:** Thus, evaluating the stage of preparation for the change in which the victims are found is important for the individual understanding of the experience and supporting the intervention. In this sense, the Domestic Violence Survivor Assessment (DVSA) will also be presented, an instrument for evaluating the process of intentional change in victims of IPV, using the TTM.

## 1. Introduction

Clinical Impact Statement: Intimate Partner Violence is one of the most common forms of violence, affecting individuals regardless of their gender, class, ethnicity, and sexual orientation, and causes several consequences for the victim in the short, medium, and long term. Both victimization and disclosure can be influenced by the quality, type, and source of social support. The request for help implies a step in the process of change, and the level of preparedness for change is directly related to the success of the therapeutic intervention in IPV.

Intimate Partner Violence: The Relationship Between the Stages of Change, Maintenance Factors, and the Decision to Keep or Leave the Violent Partner.

Intimate Partner Violence (IPV) refers to the practice of abusive behaviors between individuals who are in an intimate relationship [1,2], be it periodic or continuous acts [3]. An intimate partner is defined as an individual with whom a close/intimate relationship is maintained, characterized by the emotional connection between them, caressing, frequent contact, and knowledge of each other’s personal life, as well as identification as a couple [4]. Thus, intimate relationships are any relationships that are of a physical, emotional, or exclusively sexual nature [3]. As such, spouses, partners in a de facto union, boyfriends/girlfriends, and continuous sexual partners are considered intimate partners [4]. It is important to note that IPV can be perpetrated by current or former partners [5], as well as by partners who may or may not cohabitate [4]. So, IPV refers to behaviors by a current or former intimate partner that cause physical, sexual, or psychological consequences, and may include physical aggression, psychological abuse, and sexual coercion [6]. 

Although the term “domestic violence” (DV) is commonly used, it differs from the concept presented in this study, but there is a connection between the two [7,8]. DV pertains to violent behavior or a pattern of coercive manipulation, exercised directly or indirectly on any individual living within the same household (e.g., spouse, partner, child, father, mother, grandfather, grandmother), or towards a partner, ex-partner, or family member (in the case of non-cohabitation) [9]. Conjugal violence (CV) is a specific aspect of DV [10] and pertains to the aforementioned violent behaviors related to DV, solely committed between spouses/partners or ex-spouses/ex-partners [11].

IPV is a serious worldwide problem, persisting over several generations, and encompasses habits and customs that are legitimized by traditions and cultures, such as patriarchal ideology, as well as by the silence of the victims [12,13,14]. It has aroused the interest of several researchers, social structures, and the community in general [15,16].

IPV has been framed within policies for prevention, criminalization, and victim support [17], and nowadays it is considered a crime in several countries [18].

IPV is a complex phenomenon because there is an intense emotional component, as well as the sharing of life plans and responsibilities [11]. Therefore, the offender has information and strategies that can be used to control the victim and lead him/her to become dependent on the offender, making the process of leaving the relationship more costly [10].

It is a public health problem, as well as a social and criminal problem [4], and represents a severe violation of human rights [2,5,19]. This is a silent phenomenon, as it is a type of crime with some of the highest rates of dark figures [20].

According to various data, IPV occurs mainly from adolescence onwards [21,22,23], and is quite frequent in marital or cohabitation relationships [2].

There is a large variation in IPV prevalence, ranging from 6.1% (physical or sexual) to 28.7% (psychological) [24]. The victims are mostly women [25], which can be seen in the data contained in the 2021 Annual Internal Security Report [26], in which 74.9% of victims were women. Despite this finding, IPV also encompasses violence against men [27]. Several studies indicate similar levels of the impact of victimization between men and women [5,28].

IPV leads to several consequences for the victim in the short, medium, and long term [11,16,26,29,30], namely physical, relational [16,20,29,31,32,33,34], psychological/emotional [16,31,32,33,35], economic [20,29,33], behavioral [31], and sexual damage [2,33]. The severity of the consequences derives from the degree of proximity to the offender, the type and duration of the victimization, and the victim’s age (both chronological and developmental) [33,36]. In the most significant cases, there may be more severe consequences, such as temporary or permanent disability, or death of the victim [14,16,36,37].

## 2. Process of Change

### 2.1. Seeking Help

Intervention in IPV is extremely difficult because victims tend to only ask for help when the episodes are already at an extreme stage, that is, when they can no longer withstand the violence [38]. Although victims are aware of the problem they are facing, they believe there is no solution and that no one can help them [39]. Moreover, many victims refuse to ask for help due to fear, shame, and social isolation [31,39,40,41], as well as the fear of humiliation or discrediting by friends, family, police, and legal organizations, among others [41]. Several studies indicate that victims do not ask for help because they do not want to disturb family and friends, or because they fear repercussions and accountability for the violence [31,37,42].

The violence inflicted by partners on victims makes it impossible to preserve and create social relationships, as it isolates and weakens them [43,44]. As such, seeking help is seen as a significant step in ending abusive relationships, and disclosing the violence suffered has a significant impact on victims [39,45]. The vast majority of victims only disclose the abusive situation when they recognize the danger they are in when they recognize their partner’s behavior is not normal, or when they realize they are not able to face the situation alone [45,46]. Despite this awareness, the request for help tends to be mostly made by female victims [47,48], with male individuals tending to hide the situation they are experiencing, mainly due to the difficulties they experience in talking about the abusive episodes or not recognizing them as violence [48].

Social support networks are a resource that helps to minimize the repercussions of IPV, in which victims can obtain support to suppress their needs in day-to-day events, as well as in crises [49]. In this context, the concept of social support is defined by the presence or availability of resources the victim can access to seek help [50].

Studies indicate that the presence of an adequate social support network provides a decreased risk of developing psychological disorders such as anxiety, depression, and post-traumatic stress [51,52,53,54,55], and also provides a reduction in consequences in terms of physical health and emotional well-being [32,55,56]. Furthermore, it also reduces the propensity for suicidal ideation [51,57]. The presence of a social support network also helps the victim to acquire coping strategies that contribute to increasing the victim’s ability to find a way to solve the crisis [51,54,56,58]. Machisa et al. [44] concluded that the presence of a social support network promotes resilience in victims. Vameghi et al. [59] demonstrated that the phenomenon of IPV tends to be less frequent when there is an active social support network.

The experience of IPV and its disclosure can be influenced by factors such as the quality, type, and source of social support received [50]. The social support network consists of individuals who can help the victim, whom the victim can trust [60], and mobilizes physical, informational, social, psychological, and/or emotional resources [57,61,62]. Several authors divide social support into two dimensions: formal and informal [46,50,63].

The formal support networks include criminal police bodies (CPBs), legal services, health services, psychology services, social workers, victim care services, shelters, and victim support lines, among other services, which have organized responses to assist victims [43,45,46,50,51,63,64,65]. Victim care services are made up of professionals from different areas, which enriches the understanding of the phenomenon and leads to more appropriate intervention [31,66]. Thus, this type of support can play a fundamental role in the victim’s process of change, as long as the network is organized and structured with other resources [43,56,67,68]. Victims tend to turn to formal support networks based on factors such as the severity and frequency of violent episodes, and perceived support, among others, which helps in the process of preparing for change [56,67,68]. Some authors indicate that victims tend to avoid the formal support network, mainly because they deny the phenomenon and/or because they do not recognize the gravity of the phenomenon they are experiencing [69,70]. Some studies conducted with victims of IPV indicate that sometimes victims may experience re-victimization by specialized support services [43,44,51,56,58]. Studies such as the one by Freeland et al. [71] indicate that, in some situations, CPBs tend to minimize the abusive experience or even dissuade the victim’s attempt to file a complaint. This phenomenon was also identified in some counseling services and support organizations [71]. On the other hand, the formal support network was also considered useful and seen as a fundamental resource in the search for help [71].

Regarding the informal support network, it is made up of family members, friends, neighbors, co-workers, and organizational groups to which the victim belongs (e.g., church, sports clubs, or political, recreational, and cultural groups) [43,45,46,56,63,72,73], which may provide material and emotional responses, such as the expression of affection, empathy, concern, availability of goods, and financial support, among others, and become essential for the well-being of the victim [72]. The informal support network is a resource that should be instigated, because, in addition to providing almost immediate answers, it helps the victim feel supported and, consequently, the offender realizes the victim is not isolated [74]. Moreover, the existence of an adequate informal support network facilitates the process of ending the relationship, as it helps the victim to find strategies and consciously make the decision [75]. Studies such as the one by Sylaska and Edwards [56] reveal that most victims disclose the IPV situation to at least one member of the informal support network, as a way of mitigating the stress experienced and because they feel heard [45]. In the study by Mahapatro and Singh [58], victims revealed that the institutions to which they resorted only provided a reduction in anxious and depressive symptoms momentarily and provisionally, while the support provided by the informal support network was seen as lasting. Despite the support provided by the informal support network being the most sought after by victims, it tends to be mentioned as inadequate by several authors [46,72]. In many studies, the participants who requested help mostly did it informally [42,56,67,69,71,76].

It is important to note that, in many cases, victims only disclosed the abusive situation after the end of the relationship [40] or when the violence became recurrent and/or severe [47]. Nonetheless, studies such as the one by Machado et al. [76] showed that most victims do not make any request for help.

### 2.2. Transtheoretical Model of Change (TTM)

The Transtheoretical Model of Change (TTM) is an explanatory model of the change process, based on a feminist perspective, developed by Prochaska and DiClemente in the 1970s [77,78], and it aims to help understand, measure, and intervene in behavior change [79,80].

In the first stage, pre-contemplation, the victim has no intention of changing shortly [81,82,83,84,85] and does not recognize the existence of a problem and the associated risks [80,82,86].

In the contemplation stage, there is an awareness of the problem [80,83,84,85,87], but the victim is not committed or making an effort to solve it [81,82,88], and not setting deadlines to begin the change process [89].

In the preparation stage, the individual makes some efforts to change the behavior [81], and is committed to fulfilling his or her goal [90] by establishing a plan [80,82].

In the action stage, the victim effectively begins to act, by changing behaviors or the environment, to solve the problem [65,80,81,87,91]. Due to the possibility of relapse, this stage requires strong dedication and commitment, as well as significant commitment [65,90].

Finally, the maintenance stage encompasses the consolidation of the results achieved in the action stage and the victim strives to prevent potential relapses [65,83,89,90].

Years later, the termination stage was added, in which the behavioral changes obtained are solid enough for the victim to feel confident that the previous behaviors will not return, thus presenting a reduced possibility of relapse [91].

Using the TTM, instruments were developed to measure the stages of change, namely the University Rhode Island Change Assessment (URICA) [92]. This instrument is applied in several areas related to health and addictive behaviors, as well as in the area of IPV [93].

## 3. Contribution of the TTM in the Process of Change in Victims of IPV

Although there are few studies specifically addressing IPV, there is research that applies contributions of the Transtheoretical Model of Change (TTM) to victims of this phenomenon [94,95]. Reisenhofer and Taft [96] conducted a study analyzing the applicability of TTM in the context of IPV to provide instructions and guidance to professionals working with victims. The study concluded that professionals should indeed use the stages of change provided by the TTM as a way of evaluating the change process [96]. Similarly, Zamora et al. [95] analyzed the reports of 35 women who experienced IPV using the TTM and concluded that the stages described in the model are applicable to IPV. Catallo et al. [97] also analyzed the sequence of events that led to the disclosure of IPV episodes among 19 victims, using TTM. This study found that disclosure typically occurs after a significant event, which becomes a turning point following a series of less severe events. The decision to disclose is often influenced by a balance between the perceived risks and the potential benefits of disclosure [97]. Furthermore, the study by Catallo et al. [97] recommended that healthcare professionals should use an assessment based on the stages of change to better understand both the change process and the phenomenon of IPV disclosure.

In terms of the characteristics of each stage of change, studies have highlighted the following patterns among victims: (a) Pre-contemplation: This stage is marked by suffering and ambivalent feelings toward the violence, often characterized by minimization, normalization, or denial of the violence and excusing the offender’s behavior. Victims in this stage show low levels of anger towards the abuser and may not yet recognize the severity of the situation [26,65,98,99]. They may also rationalize the abuse or feel it is not severe enough to warrant action. (b) Contemplation: In this stage, victims begin to recognize the abusive behavior and experience higher levels of anger. They attribute their suffering to the violence and may start to consider ending the relationship. However, they often still experience conflicting emotions toward the abuser, expressing feelings of loyalty or attachment. For instance, victims might hesitate to leave due to marital commitments or fear of the consequences [98]. There is a growing awareness of the problem, but they may still be uncertain about taking steps to address it [99]. (c) Preparation: Victims in this stage typically begin developing a strategy to end the relationship, often seeking support or gathering resources [98]. However, they often face significant difficulties in determining how to proceed and experience high levels of stress. These difficulties can include uncertainty about how to leave safely, fears of retaliation, or financial instability [82]. (d) Action: This stage is marked by concrete efforts to change the situation. Victims may end the relationship, seek legal protection, or find safety in shelters. Significant behavioral and environmental changes take place, including contacting support services or confiding in trusted individuals [99]. It is a critical stage, but it can also involve the risk of relapse or re-engagement with the abuser. (e) Maintenance: After ending the abusive relationship, victims work to maintain the changes achieved and prevent relapse. This phase may involve managing ongoing emotional and psychological difficulties, such as depression, anxiety, and discouragement about the future. However, victims may also experience an expansion of social networks and increased support [99]. In this stage, long-term coping mechanisms and ongoing psychological support are crucial for maintaining a safe and healthy life post-violence.

The research highlights that these stages are not always linear; victims may move between stages or experience multiple stages simultaneously, depending on the circumstances and the support available to them.

## 4. Domestic Violence Survivor Assessment (DVSA)

The Domestic Violence Survivor Assessment (DVSA) [100], which is being validated for Portugal by the authors of the present work, is an instrument that, through the TTM, aims to assess the process of intentional change among IPV victims [101]. This instrument was developed by researchers, with the collaboration of psychologists and victim support professionals, to obtain a better understanding of the victims’ cognitive status to support them during the follow-up process, helping them to solve their dilemmas [100]. The questions developed in this instrument are based on the subjective change process of each victim, examining their perceptions about the violent relationship with their partners and how they interrupt or stop the perpetuation of violence [101].

The assessment process for the effectiveness of the DVSA indicated its usefulness for the systematic review of the difficulties that most victims of IPV face, aiding in the beginning of the follow-up [100]. The DVSA was also applied in the Montgomery County Maryland Abused Persons Program, to evaluate the results of the individual psychotherapeutic follow-up of 355 women victims of IPV [102]. This program maintains the use of DVSA and indicates it is a very useful resource in measuring the impact of follow-up [102].

In the process of validating the original scale, the professionals who applied this instrument reported it was relevant both for victims who are in abusive relationships and for victims who have already abandoned the relationship, but who continue to be monitored [101]. They reported that the scale is useful for identifying areas that need more attention in the intervention, as well as for victims to obtain the validation that their experiences in the change process are common and that change is a prolonged and lasting process [101].

DVSA contains questions designed to assess aspects of the change process as women victims shift their perspective on their abusive relationships and take steps to end the abuse. The final version consists of twelve questions, six of which are related to changes in the abusive relationship and are referred to as “relationship questions”. The remaining six questions focus on changes within the women themselves and are referred to as “individual questions” [101].

The relationship questions address the following aspects: triggers that initiate abusive episodes; behaviors adopted to manage these episodes (both direct and indirect); awareness of the need for legal support; attachment to the relationship (attachment to the partner, social value); perspectives on the relationship and available options; management of loyalty and personal beliefs (social stigma and values related to staying or leaving the relationship).

The main individual questions include access to help (the belief that family, friends, and organizations can provide assistance); self-identity (separation of the couple’s identity); self-confidence; emotional responses to abuse (level of awareness and control over emotions); mental health, stress, depression, and PTSD (Post-Traumatic Stress Disorder); and control over finances.

## 5. Discussion and Conclusions

The objective of this work was to present a comprehensive review of IPV and the change process.

As seen, IPV constitutes one of the most common forms of violence, preserved throughout generations, being grounded in habits and practices that are legitimized by traditions and cultures, and maintained by the silence of the victims [12,13,14,103], IPV generates extreme consequences in the short, medium, and long term [16,29,104].

The existence of an intense emotional component [11] allows the aggressor to arrange information and strategies that are used as a form of control and dependence on the victim, strategies that are demonstrated in PCW [105,106]. These strategies make the process of breaking up the relationship more difficult [10]. Therefore, understanding these strategies and the dynamics associated with the IPV that support the maintenance of the abusive relationship become important for intervention with victims [11]. However, it must be borne in mind that intervention with victims becomes difficult due to the time gap between the occurrence of the first abusive episodes and the search for help [38], this being a step with a significant impact that also leaves a mark on the change process [39,45].

Furthermore, involvement in the change process is an indicator of a successful intervention with victims [35], and some investigations apply TTM contributions to victims of IPV, e.g. [26,95,96,97]. Thus, it is important to identify the stage in which the victim is, to adapt the intervention to their needs [93,107].

For this, the instruments for evaluating the stages of change become useful for the effectiveness of the intervention. Given the need for instruments for this phenomenon, Dienemann et al. [100] developed the DVSA, an instrument that provides this assessment, having recognized its usefulness for the systematic review of the difficulties and dilemmas by which most victims pass [101], and, for this reason, it provides great support in the intervention process.
